# Organophosphorus Polyurethane Ionomers as Water Vapor Permeable and Pervaporation Membranes

**DOI:** 10.3390/polym13091442

**Published:** 2021-04-29

**Authors:** Ilsiya M. Davletbaeva, Oleg O. Sazonov, Ilyas N. Zakirov, Askhat M. Gumerov, Alexander V. Klinov, Azat R. Fazlyev, Alexander V. Malygin

**Affiliations:** 1 Department of Synthetic Rubber, Kazan National Research Technological University, 68 Karl Marx st., Kazan 420015, Russia; zakirovilyas1996@gmail.com (I.N.Z.); gumerov_a@mail.ru (A.M.G.); 2 Department of Chemical Process Engineering, Kazan National Research Technological University, 68 Karl Marx st., Kazan 420015, Russia; alklin@kstu.ru (A.V.K.); fazlyev.azat@gmail.com (A.R.F.); mav@kstu.ru (A.V.M.)

**Keywords:** pervaporation membranes, polyurethane ionomers, dehydration, water vapor permeability, isopropanol

## Abstract

Organophosphorus polyurethane ionomers (AEPA-PU) based on aminoethers of *ortho*-phosphoric acid (AEPA) were obtained and studied as pervaporation membrane materials for separating isopropanol/water mixtures. The regularities of the change in the water vapor permeability of AEPA-PU were also investigated. It has been established that an increase of solute content in the composition of the urethane-forming system and the content of ionogenic groups in AEPA leads to a noticeable increase in the vapor permeability of the resulting film materials. An increase in water vapor permeability values is accompanied by a significant increase in the pervaporation characteristics of AEPU-PU. It was shown that the conditions promoting clustering of phosphate anions cause an increase in the values of the vapor permeability coefficient of AEPA-PU obtained using polyoxypropylene glycol. However, the hydrophobicity of the polypropylene glycol surrounding the clusters makes it difficult for water to move through the polymer matrix. Due to the hydrophilicity of polyoxyethylene glycol, the highest values of water vapor permeability and pervaporation characteristics are achieved for AEPA-PU synthesized using PEG.

## 1. Introduction

Pervaporation separation using polymer membranes is promising in the processes of isopropanol (IPA) dehydration [1,2,3,4]. It is known that distillation methods traditionally used for these purposes have high-energy consumption and have limitations in the separation of azeotropic mixtures [5]. The use of traditional separation methods, in this case, is economically impractical and leads to environmental pollution. Isopropanol is widely used in modern semiconductor and microelectronic industries as a solvent and cleaning agent, the extraction and purification of natural products, as a binding agent, polymerization modifier, deicing agent and preservative, and as an aerosol solvent in medicine. The demand for IPA is growing every year, which contributes to an increase in the price of the pure substance. In addition, IPA waste is extremely harmful to the environment and requires high disposal costs. Therefore, the recovery and reuse of IPA are important from both economic and environmental points of view. The main problem in separating IPA from wastewater is the formation of an azeotropic mixture, which consists of 87.4 wt.% IPA and 12.6 wt.% water.

Pervaporation membranes have been developed from several hundred types of polymeric materials [6,7,8,9,10,11,12,13,14,15,16,17,18,19,20]. However, polymer membranes are limited by their poor resistance to pollution, poor chemical and thermal stability, and, in particular, by the problem of maintaining a balance between vapor permeability and selectivity. Thus, the development of new polymer membranes has become an urgent task.

Pervaporation membranes are promising new options for use as polyurethanes [21,22,23,24,25,26,27,28,29,30,31]. The selectivity and productivity of PU membranes are influenced by the chemical and physical structure of membrane materials, particularly the ratio between soft and hard segments and the degree of crosslinking. Nevertheless, few publications are devoted to the use of PU membranes for the pervaporation separation of isopropanol/water mixtures [21,22,25,26]. In this way, the effect of the NCO/OH ratio on the characteristics and selectivity of polyurethane membranes during the pervaporation separation of isopropanol/water and ethanol/water mixtures was investigated [21]. Polyurethane membranes with a thickness of 90–100 µm were obtained in two stages. First, hydroxyl-terminated polybutadiene (HTPB) was polymerized with 4,4-dicyclohexylmethane diisocyanate (H_12_MDI) with terminal –NCO groups. Then, upon reaching the theoretically calculated value of the -NCO groups, the polymer chain was extended with 1,4-butanediol (1,4-BD). The authors have shown the influence of the NCO/OH ratio on the transport properties of PU membranes. The best pervaporation characteristics during dehydration of isopropanol (10 wt.% water) were achieved for NCO/OH = 1.5 at a temperature of 30 °C (320 g/m^2^·h, selectivity—180).

In [22], polyurethanes were modified by immersing untreated PU membranes in a mixture of formic acid and hydrogen peroxide with a molar ratio of 1:1. Further, 90 µm thick polyurethane membranes based on HTPB, H_12_MDI, and 1,4-BD were synthesized using the same two-step method. The authors showed that an increase in the time of PU membranes epoxidation leads to improvement in performance during the pervaporation dehydration of isopropanol. The formation of epoxy and ether groups on the membrane surface increases the affinity of isopropanol for the membrane, which is reflected in the separating properties of epoxidized polyurethane membranes. The best pervaporation characteristics during the dehydration of isopropanol (10 wt.% water) were achieved at a temperature of 25 °C (1583 g/m^2^·h, selectivity—4.67).

Hydrophilic polyurethanes are most often used in pervaporation processes, but their use is limited by excessive swelling, which leads to a deterioration in mechanical properties and separation characteristics. Tsai et al. [25] proposed increasing the resistance of the polyurethane membrane to solvents and pervaporation characteristics due to spatial crosslinking with methyl methacrylate (MMA). In this work, PU membranes based on HTPB, H12MDI, and 1,4-BD with a thickness of 90–100 µm were synthesized in two stages. Then crosslinking was performed by sequential polymerization at HTPB/MMA ratios (wt./wt.% ratio) = 2.0, 1.5, 1.0, 1.5, 0.8, and 0.6., which were designated as IPN1–IPN5, respectively. The best performance (365 g/m^2^·h) and selectivity (212) in comparison with the standard PU in the pervaporation separation of isopropanol (10 wt.% water) at an operating temperature of 30 °C was shown by the composition IPN3; this is explained by the fact that the crosslinking of PU with MMA leads to an increase in the intermolecular space and, consequently, to an increase in transport characteristics.

In [26], an efficient polyurethane membrane was developed for the separation of methanol/water, ethanol/water, and isopropanol/water mixtures based on polyethylene glycol (PEG) with different molecular weights and 2,4-toluene diisocyanate. The molecular weight of the polyether has a significant effect on the selectivity and productivity of polyurethane membranes. The developed polyurethane membrane based on PEG-4000 has a good potential for separating an isopropanol/water mixture (20 wt.% water) at a temperature of 30 °C, demonstrating a productivity of 730 g/m^2^·h and a selectivity of 13.

The main disadvantages of existing polyurethane membranes are that it is difficult to maintain a balance between performance and selectivity and low chemical and mechanical stability. Promising for obtaining highly selective and efficient pervaporation membranes are polyurethane ionomers (PUI) [32,33,34,35,36,37,38,39], which can form highly efficient conducting channels for water molecule transport due to the ability of clustering of ionogenic groups.

**Figure 1:**
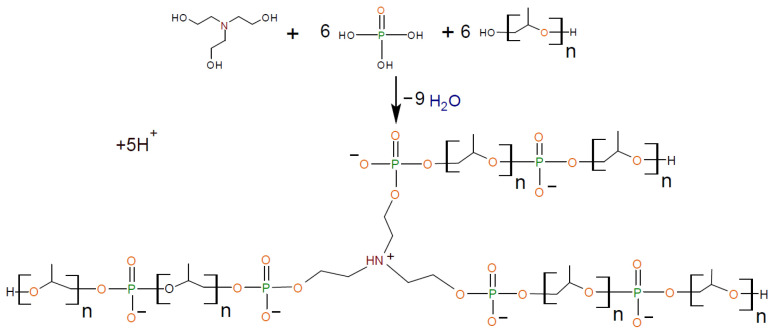
Formation of AEPA-6-PPG.

In [40,41], exhibiting thermosensitive water vapor permeability, porous polyurethane ionomers based on the aminoethers of boric acid were synthesized. In [42,43], polyurethane ionomers (AEPA-PPG-PU) were obtained based on aminoethers of *ortho*-phosphoric acid (AEPA-PPG). It was found that, during the preparation of AEPA-PPG using triethanolamine as a catalyst and a branching center, a branched compound is formed, in which, due to incomplete etherification of H_3_PO_4_ with triethanolamine and polyoxypropylene glycol (PPG), phosphate anions are present and surrounded by protons (Figure 1).

**Figure 2:**
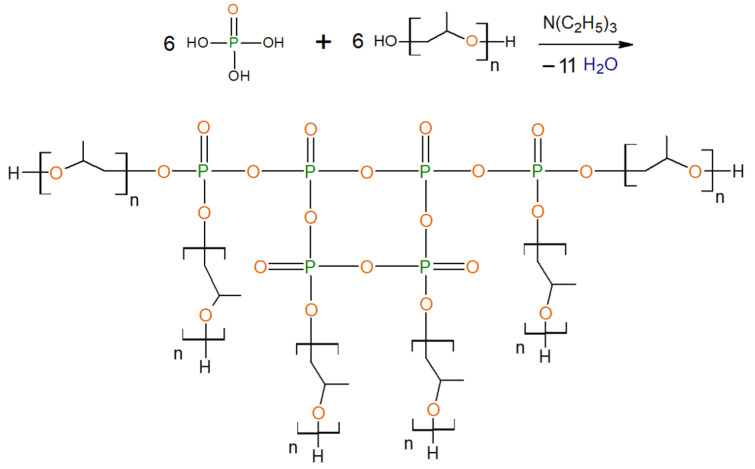
Formation of EPA-6-PPG.

In the case of the etherification of H_3_PO_4_ using triethylamine as a catalyst, branched compounds (EPA) are also formed (Figure 2), but the H_3_PO_4_ undergoes complete etherification with PPG and the formation of polyphosphate structures. There are practically no ionogenic groups in these compounds.

The ionomeric nature of AEPA-PU was the reason for their study as pervaporation membrane materials for the separation of water–alcohol mixtures. To change the content of ionogenic groups in the composition of AEPA and influence the supramolecular organization of aminoethers of *ortho*-phosphoric acid, polypropylene glycol with MW = 400, 1000, 2000 (PPG-400, PPG-1000, PPG-2000), and polyoxyethylene glycol with MM = 400 (PEG-400) were used during synthesis.

## 2. Materials and Methods

### 2.1. Solvents and Reagents

Polyoxypropylene glycol (PPG-400/1000/2000) and polyoxyethylene glycol (PEG-400) were purchased from Wanhua Chemical (Beijing, China). Triethanolamine (TEOA) and triethylamine (TEA) were obtained from Ltd. “Component-reaktiv” (Moscow, Russia). Toluene was obtained from Ltd. «Component-reaktiv» (Moscow, Russia). An 85% aqueous solution of *ortho*-phosphoric acid (OPA) was purchased from Ltd. «MCD-Chemicals» (Moscow, Russia). 2-Methylimidazole was purchased from «Acros Organics BVBA» (Geel, Belgium). Polyisocyanate “Wannate PM-200” (PIC) was purchased from Kumho Mitsui Chemicals, Inc. (China). Isopropyl alcohol (Propanol-2) was purchased from CJSC «Synthetic Spirit Plant» (Orsk, Russia).

### 2.2. Synthetic Procedures

#### 2.2.1. General Procedure for Synthesis of Amino Ethers of Ortho-Phosphoric Acid (AEPA-PEG-400)

The etherification reaction was carried out in one stage by reacting H_3_PO_4_ with TEOA and PEG-400. To obtain AEPA, triethanolamine, *ortho*-phosphoric acid, and PPG-1000 were used at their molar ratios [TEOA]:[H_3_PO_4_]:[PEG-400] = 1:3:6, 1:4:6, 1:5:6, 1:6:6 (AEPA-3-PEG-400, AEPA-4-PEG-400, AEPA-5-PEG-400, and AEPA-6-PEG-400, respectively). The calculated amount of H_3_PO_4_ and PEG-400 was placed in a round-bottom flask, mixed for two minutes, then TEOA was added to the reaction system. Within two hours, the reaction mass was stirred at T = 80 °C and a residual pressure of 0.7 kPa. The synthesized liquid AEPA-PEG-400 was collected into a stoppered flask. The amount of residual water did not exceed 0.2 wt.%.

#### 2.2.2. General Procedure for Synthesis of Amino Ethers of Ortho-Phosphoric Acid (AEPA-(3 ÷ 6)-PPG-400/1000/2000)

The etherification reaction was carried out in one stage by reacting OPA with TEOA and PPG. To obtain AEPA, triethanolamine, *ortho*-phosphoric acid and PPG-400/1000/2000 were used at their molar ratios [TEOA]:[H_3_PO_4_]:[PPG] = 1:3:6, 1:4:6, 1:5:6, 1:6:6 (AEPA-3-PPG-400/1000/2000, AEPA-4-PPG-400/1000/2000, AEPA-5-PPG-400/1000/2000, and AEPA-6-PPG-400/1000/2000, respectively). The calculated amount of H_3_PO_4_ and PPG was placed in a round-bottom flask, mixed for two minutes, and then TEOA was added to the reaction system. Within two hours, the reaction mass was stirred at T = 80 °C and a residual pressure of 0.7 kPa. The synthesized liquid AEPA-PPG-400/1000/2000 was collected into a stoppered flask. The amount of residual water did not exceed 0.3 wt.%.

#### 2.2.3. General Procedure for Synthesis of Ethers of Ortho-Phosphoric Acid (EPA-(3 ÷ 6)-PPG-1000)

The etherification reaction was carried out in one stage by reacting H_3_PO_4_ with TEA and PPG. To obtain EPA, triethylamine, *ortho*-phosphoric acid and PPG-1000 were used at their molar ratios [TEA]:[H_3_PO_4_]:[PPG-1000] = 1:3:6, 1:4:6, 1:5:6, 1:6:6 (EPA-3-PPG-1000, EPA-4-PPG-1000, EPA-5-PPG-1000, and EPA-6-PPG-1000, respectively). The calculated amount of H_3_PO_4_ and PPG-1000 was placed in a round-bottom flask, mixed for two minutes, and then TEA was added to the reaction system. Within two hours, the reaction mass was stirred at T = 80 °C and a residual pressure of 0.7 kPa. The synthesized liquid EPA-(3 ÷ 6)-PPG-1000 was collected into a stoppered flask. The amount of residual water did not exceed 0.3 wt.%.

#### 2.2.4. General Procedure for Synthesis of Polyurethanes Based on Amino Ethers of Ortho-Phosphoric Acid and Ethers of Ortho-Phosphoric Acid (AEPA-(3 ÷ 6)-PPG-1000/2000-PU, EPA-(3 ÷ 6)-PPG-1000-PU, AEPA-PEG-400-PU).

The synthesized AEPA-(3 ÷ 6)-PPG-1000/2000, EPA-(3 ÷ 6)-PPG-1000, and AEPA-(3 ÷ 6)-PEG-400 were mixed with PIC in specific ratios (1:1). Then, the solvent (toluene) was added in a certain amount to obtain a urethane-forming reaction system with a certain solute content (SC). Stirring was continued for 5 min at room temperature and the mixture was cast onto the prepared surfaces to form polyurethane films. After the solvent evaporated, the curing of polyurethanes was carried out for 24 h at room temperature. After the final curing, the samples were heated for 10 min at 100 °C to remove residual solvent. As a result, film materials were obtained under conditions of solute content from 20 to 100 wt.% (SC = 20 ÷ 100 wt.%).

### 2.3. Manufacturing of Pervaporation Membranes

A hydrophilic porous support based on a fluoroplastic—MDK (composite membrane)-1,2 (pore size 500 nm, thickness 90 microns, from Vladipor, Russia)—was chosen to prepare the supported membranes with AEPA-PPG-1000-PU and a hydrophilic porous support based on a fluoroplastic—UFFK (ultrafiltration membranes) (pore size 50 nm, thickness 90 microns, from Vladipor, Russia)—was chosen to prepare the supported membranes with AEPA-PEG-400-PU. The main reason for using different substrates is that AEPA and polyurethanes synthesized from them are obtained from different natures of polyether and have different wetting properties.

To manufacture the composite membrane, the selective layer was applied to the substrate using a cylindrical applicator KAU 1 (Constanta, St. Petersburg, Russia). The substrate was fixed to a glass plate to prevent any folding or twisting. The thickness of the applied selective layer was set using guides of constant thickness fixed at the edges of the substrate, on the surface of which the applicator was manually moved. The selective layer was cured at room conditions for 24 h.

### 2.4. Fourier Transform Infrared Spectroscopy Analysis (FTIR)

The FTIR spectra of the products were recorded on the Nicolet iS20 FT-IR spectrometer (Thermo Fisher Scientific, Waltham, MA, USA) using the attenuated total reflection technique. The spectra were acquired by accumulating 64 scans at a spectral resolution of 4 cm^−1^ in an absorbance mode from 3600 to 600 cm^−1^.

### 2.5. Viscosity and Density Measurements

The dynamic viscosity of the samples was determined at the T = 20 °C and atmospheric pressure on an SVM 3000 Stabinger Viscometer (Anton Paar, Austria), with a systematic error of ± 0.35% of the measured value. At the same time, the density of the samples was determined with a systematic error of 0.0005 g/cm^3^.

### 2.6. Measurements of the Surface Tension

The droplet counting method was used to determine the surface tension (σ). The basis of the calculations is the equation, where the weight of the drop that comes off the pipette is proportional to the surface tension of the fluid and the radius of the pipette (R): m = 2π·R·σ/g, where: *g* is the acceleration of gravity; *m* is the drop mass of the test liquid. Following this equation: σ = mg/2πR. Further, according to the obtained results, the surface tension (σ) was constructed from the characteristic curve of the concentration (C).

### 2.7. Tensile Stress–Strain Measurements

Tensile stress–strain measurements were obtained from the film samples of size 40 mm × 15 mm with Universal Testing Machine Inspekt mini (Hegewald&PeschkeMeß- und Prüftechnik GmbH, Nossen, Germany) at 293 ± 2 K, 1 kN. The crosshead speed was set at 50 mm/min, and the test continued until sample failure. Minima of five tests were analyzed for each sample, and the average values were reported.

### 2.8. Thermomechanical Analysis (TMA)

The thermomechanical curves of polymer samples were obtained using TMA 402 F (Netzsch, Selb, Germany) thermomechanical analyzer in the compression mode. The sample thickness was 2 mm, and the rate of heating was 3 °C/min from −50 °C to 350 °C in the static mode. The load was 2 N.

### 2.9. Mechanical Loss Tangent Measurements (MLT)

The MLT curves of polymer samples were taken using the dynamic mechanical analyzer DMA 242 (Netzsch, Selb, Germany) in the mode of oscillating load. Force and stress–stain correspondence was calibrated using a standard mass. The thickness of the sample was 2 mm. Viscoelastic properties were measured under nitrogen. The samples were heated from −50 °C to 350 °C at the rate of 3 °C/min and a frequency of 1 Hz. The mechanical loss tangent was defined as the ratio of the viscosity modulus G″ to the elasticity modulus G′.

### 2.10. Thermal Gravimetric Analysis (TGA)

TGA was performed using STA-600 TGA–DTA combined thermal analyzer (Perkin Elmer, Waltham, MA, USA). The samples (0.1 g) were loaded in alumina pans and heated from 30 to 750 °C at a rate of 5 °C/min in a nitrogen atmosphere.

### 2.11. The Densitometer

The density of the composites was determined on an H-300S (Hildebrand, Oberboihingen, Germany) densitometer with an ultra-high resolution of 0.001 g/cm^3^.

### 2.12. Water Adsorption

Water adsorption was determined by the gravimetric method. A sample of a certain size was cut out from the obtained polymer. The resulting sample was weighed on an analytical balance. The measuring cup loaded with distilled water, and then the sample was placed in water and fixed. After a specified amount of time, the samples were removed, the remaining water from the surface was removed using filter paper, and the sample was weighed. Water adsorption (B) is calculated as a percentage using the formula:

B = (ms − m)/m(1)

where: m—the mass of the sample before the test, g; ms—the mass of the sample after the test. For the result, the arithmetic average of the results of the 5 tests was used.

### 2.13. Water Vapor Permeability (WVP) Measurements

WVP was measured according to the ASTM method of E 96–80B. Round mouth cylindrical glass cups with a diameter of 50 mm and a height of 70 mm were filled with deionized water. Membranes were placed over the top of the cups and secured the perfect sealing between the cup and the membranes. The gaps between the membrane and the water surface were about 15 mm. The cups were placed in a constant temperature chamber at the temperature 22 °C and 40 °C. The humidity in the chamber was 90%. The weight losses after 24 h were measured. The result of water vapor permeability was calculated by the following formula:

(2) $$WVP=\frac{G}{tA}$$

where *G* is the weight change in grams; *t* is the duration of the test in hours; *A* is the test area in m^2^.

During all WVP measurements, the air surrounding the membranes had a constant temperature and relative humidity of 90%. Sample thicknesses for all measurements were in the range of approximately 80 microns. On average, three different readings were used for each WVP measurement, which were expressed in units of g/m^2^ 24 h.

### 2.14. Pervaporation Processes

**Figure 3:**
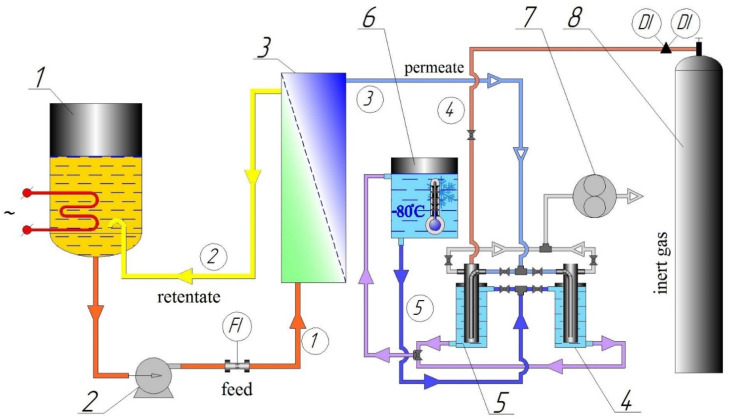
Scheme of the experimental pervaporation unit: 1—raw material capacity; 2—feed pump; 3—cell for membranes; 4, 5—cold traps; 6—cryostat; 7—vacuum pump; 8—inert gas cylinder.

Experimental studies were carried out for an isopropanol/water mixture at a concentration of 85 wt.% isopropanol and 15 wt.% water in a model mixture, at a temperature of 60 °C/40 °C, and a vacuum depth of 20 mm Hg. Art. A model mixture of isopropanol/water was prepared from demineralized water (specific conductivity of 5 μS/cm) obtained on an Osmodemi 12 setup (ERREDUE S.P.A, Livorno, Italy) and dehydrated isopropanol with a main component content of 99.8 wt.%. A scheme of the experimental pervaporation unit used to study the separation characteristics of polymer membranes is shown in Figure 3.

The plant consists of two main parts—raw material and vacuum (permeate) part. In the raw part, with the help of a pump through lines 1 and 2 (line numbers are enclosed in a circle), the raw material is circulated between the raw material tank and the membrane cell. The pressure in the raw material is close to atmospheric. A thermal cable is fixed on the wall of the raw material tank to maintain the set temperature of the experiment. In the cell for membranes, the raw material moves in a spiral along the surface of the membrane at a speed of more than 2 m/s; this makes it possible to reduce the concentration polarization. The vacuum under the membrane is created using a membrane-type vacuum pump. From the casing of the membrane module, permeate vapors, moving along line 3, fall into cold flow traps 4 or 5, which are cooled by a refrigerant with a temperature of −80 °C supplied through line 5 from the cryostat. Cold trap 4 is a prelaunch one and is used to bring the installation to a given mode; the experiments themselves were carried out on trap 5, which is a measuring one. After the end of the experiment, measuring trap 5 was cut off from line 3 and the line of connection with vacuum pump 6. To equalize the pressure and prevent moisture condensation from the air, nitrogen is supplied to the trap 5 through pipeline 4.

The composition permeate flux was analyzed using gas chromatograph Crystal-2000M (JSC SDO «Chromatec», Yoshkar-Ola, Russia), which was equipped with a thermal conductivity detector and with a capillary column HP-FFAP 50 m × 0.53 mm × 0.25 μm (Agilent Technologies, Inc., Santa Clara, CA, USA). Helium was the carrier gas, with a total of 20 mL/min. Samples of 1 μL were injected with a liquid autosampler AS-2M SP (JSC SDO «Chromatec») into the chromatograph. The injection port temperature, detector, and column were equal to 220 °C, 250 °C, and 77 °C, respectively. Samples were analyzed using the Chromatec Analytic software (version 2.6.014, JSC SDO «Chromatec»).

The values of the total permeate flux $\bar{J},$ the separation factor α, and the pervaporation separation index PSI were determined from the measurement data using the following expressions:

(3) $$\bar{J}=\frac{m^{p}}{F\Delta t}\;\alpha =\frac{{\bar{x}}_{A}^{P}/{\bar{x}}_{A}^{F}}{{\bar{x}}_{B}^{P}/{\bar{x}}_{B}^{F}}\;\alpha =\frac{{\bar{x}}_{A}^{P}/{\bar{x}}_{A}^{F}}{{\bar{x}}_{B}^{P}/{\bar{x}}_{B}^{F}}$$

where $m^{p}$ is the mass of the permeate, kg, collected over the time interval Δt, h.; F is the membrane surface area m^2^; ${\bar{x}}_{A}^{P}$ and ${\bar{x}}_{B}^{P}$ are the mass concentrations of the components A (isopropanol) and B (water) in the permeate, respectively, wt.%; ${\bar{x}}_{A}^{F}$ and ${\bar{x}}_{B}^{F}$ are mass concentrations of components A and B in retant (raw material), respectively, wt.%.

## 3. Results

### 3.1. Membranes Based on AEPA-PPG-1000/2000-PU

#### 3.1.1. Water vapor permeability of AEPA-PPG-1000/2000-PU

Film samples of AEPA-PPG-1000-PU were obtained both by casting them from solutions of the urethane-forming system and directly from the SC = 100 wt.%, i.e., without using a solvent.

**Figure 4:**
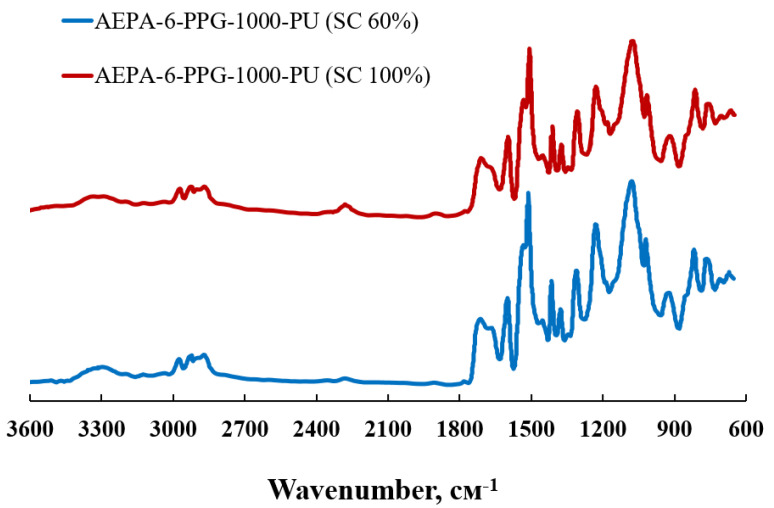
FTIR spectra of AEPA-6-PPG-1000-PU obtained at different SC.

The completeness of the terminal hydroxyl groups’ interaction of the AEPA with the isocyanate groups of the PIC can be judged by the FTIR spectra analysis. The spectra do not show the characteristic bands at 2275 cm^−1^ of NCO groups (Figure 4).

**Figure 5:**
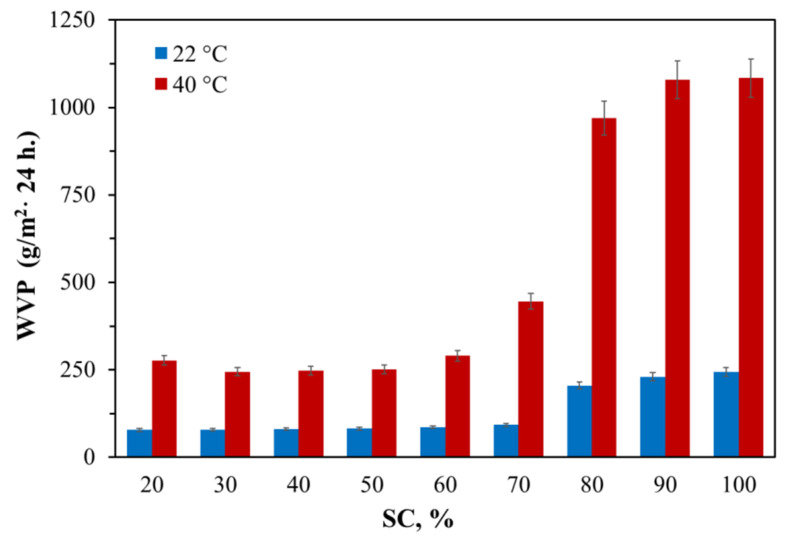
Water vapor permeability coefficients for AEPA-6-PPG-1000-PU obtained at different SC.

It was found that the solute content (SC) in the solutions of the urethane-forming system has a significant effect on the water vapor permeability of the resulting film materials. AEPA-6-PPG-1000-PU obtained at SC = 60 wt.% exhibit a low degree of water adsorption (1.2 wt.%) and low vapor permeability (Figure 5). Starting with an SC content of 70 wt.%, the water vapor permeability begins to increase markedly, increasing 2.5 times at SC = 100 wt.%. At the same time, an increase in water adsorption (3.3 wt.%) of AEPA-6-PPG-1000-PU is observed.

Due to the fact that the highest values of water adsorption and water vapor permeability are observed at SC = 100 wt.% in the solutions of the urethane-forming system, further studies of vapor permeability and pervaporation characteristics were carried out for polyurethanes obtained at SC = 100 wt.%.

**Figure 6:**
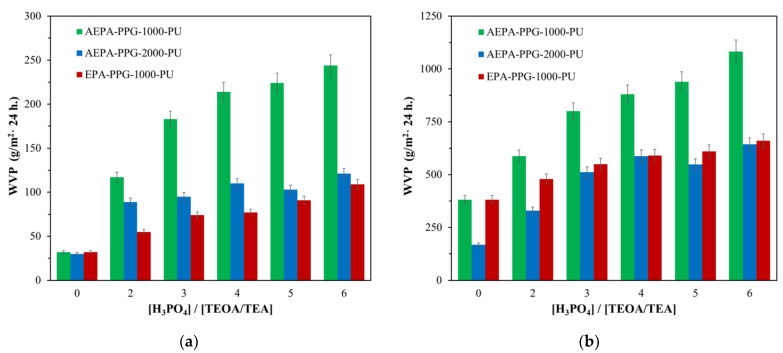
Dependences of the water vapor permeability coefficients at 22 ℃ (**a**) and 40 ℃ (**b**) on the molar ratio of [H_3_PO_4_]/[TEOA] for AEPA-PPG-1000/2000-PU and [H_3_PO_4_]/[TEA] for EPA-PPG-1000-PU, obtained at SC = 100 wt.%.

The water vapor permeability of the samples is significantly influenced by the content of H_3_PO_4_ in the AEPA-PPG-1000-PU composition (Figure 6). An increase in the content of H_3_PO_4_ in the composition of AEPA-PPG-1000 and, accordingly, in AEPA-PPG-1000-PU leads to an increase in the content of ionogenic groups in the composition of AEPA-PPG-1000-PU.

To substantiate the key role of ionogenic groups in the formation of water vapor permeability of AEPA-PPG-1000-PU, polyurethanes of non-ionic nature (EPC-PPG-1000-PU) obtained based on EPA were studied. According to Figure 6, the water vapor permeability of EPA-PPG-1000-PU also increases with an increase of H_3_PO_4_ content in the composition of EPA. However, the values of the water vapor permeability coefficient themselves turned out to be more than two times lower in comparison with the samples of AEPA-PPG-1000-PU. It should be noted that the water adsorption values for EPA-6-PPG-1000-PU obtained at SC = 100 wt.% are slightly lower (2.1 wt.%) in comparison with AEPA-6-PPG-1000-PU obtained under similar conditions (3.3 wt.%).

In order to confirm the decisive role of the supramolecular structure on the vapor permeability of thin films of AEPA-PPG-1000-PU, polyurethanes were synthesized using PPG with MW = 2000 (AEPA-PPG-2000-PU). The premise for this approach was the assumption that a higher molecular weight of PPG-2000 molecules in comparison with PPG-1000 entails a corresponding decrease in the content of ionomeric structures in AEPA-PPG-2000-PU in comparison with AEPA-PPG-1000-PU. According to Figure 6, an increase in the molecular weight of PPG led to a decrease in the water vapor permeability coefficient for AEPA-PPG-2000-PU relative to AEPA-PPG-1000-PU. The water adsorption values for AEPA-5-PPG-2000-PU and AEPA-6-PPG-2000-PU remained low (2.1 and 1.9 wt.%).

#### 3.1.2. Surface-Active Properties of AEPA-PPG-1000/2000

**Figure 7:**
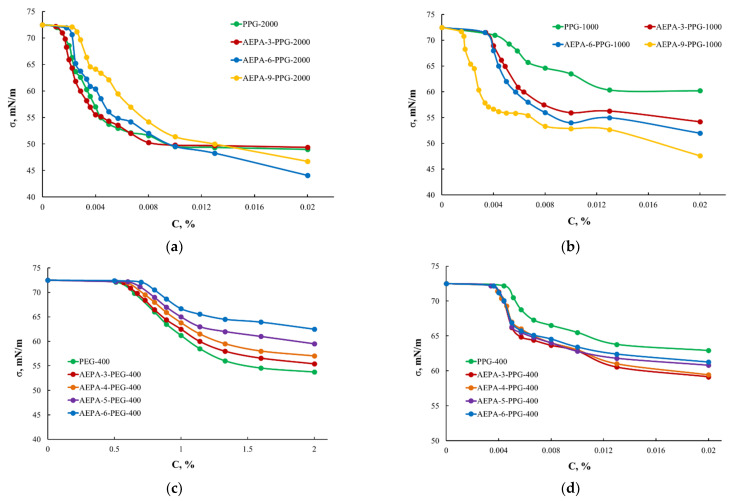
Surface tension isotherms for AEPA-PPG-2000 and PPG-2000 (**a**); AEPA-PPG-1000 and PPG-1000 (**b**); AEPA-PEG-400 and PEG-400 (**c**); AEPA-PPG-400 and PPG-400 (**d**).

In connection with the results obtained, comparative studies of the surface tension of AEPA-PPG-1000 and AEPA-PPG-2000 were carried out. According to Figure 7a, the critical micelle concentration and the surface tension of PPG-2000 turned out to be lower than these values measured for PPG-1000. Nevertheless, the regularities of changes in surface tension for AEPA-PPG-2000 and AEPA-PPG-1000 are similar in manifestation, i.e., the structure of AEPA-PPG-2000, similarly to AEPA, is ionomeric, the only difference is in the length of the polyether branches.

#### 3.1.3. Study of AEPA-PPG-1000-PU as Pervaporation Membranes for the Separation of Isopropanol/Water Mixtures

Studies of the regularities of the vapor permeability coefficient changes made it possible to establish that the WVP values noticeably increase both with an increase in the content of ionogenic groups in the composition of AEPA-PPG-1000-PU and with an increase in the content of SC in the urethane-forming system. AEPA-PPG-1000-PU, obtained at SC = 100 wt.%, was used to manufacture pervaporation membranes for isopropanol dehydration. According to the results shown in Table 1, polyurethane membranes exhibit high membrane performance, which increases with an increase in the content of ionogenic groups in the composition of AEPA-PPG-1000-PU.

In order to evaluate the advantages of the obtained pervaporation membranes, in the introductory part of this article, the indicators of the separation efficiency of the isopropanol/water mixture of the currently known pervaporation polyurethane membranes were given. So, in [21], polyurethanes were obtained on the basis of oligobutadienediol, 4,4-dicyclohexylmethane diisocyanate, and 1,4-butanediol. The highest flow values for the isopropanol/water mixture was 320 g/m^2^·h, and the selectivity was 180. In this case, testing was carried out at an isopropanol content of 90 wt.% and operating temperature of 30 ℃. In [22], epoxidized polyurethane membranes were synthesized on the basis of oligobutadienediol, 4,4-dicyclohexylmethanediisocyanate, and 1,4-butanediol. The highest productivity of epoxidized polyurethane membranes for an isopropanol/water mixture at 25 ℃ was 1583 g/m^2^·h, and the selectivity was 4.67. In [25], pervaporation polyurethane membranes with an interpenetrating polymer network, obtained on the basis of oligobutadienediol and polymethyl methacrylate, were studied. When separating a 90 wt.% isopropanol solution, the highest productivity of such membranes was 365 g/m^2^·h, and the selectivity was 212 at an operating temperature of 30 ℃. In [26], the authors investigated a number of polyether urethane membranes based on polyoxyethylene glycol and 2,4-toluene diisocyanate by the pervaporation method for isopropanol/water mixtures. The authors stated that the selectivity and performance of polyurethane membranes are significantly influenced by the molecular weight of the polyether. A membrane based on PEG-4000 showed the highest efficiency in separating of isopropanol/water mixture. At an operating temperature of 30 ℃ and water content of 80 wt.% in the separated mixture, the flow values were 481.9 g/m^2^·h, and the selectivity was 68.7. At a water content of 20 wt.% in the separated mixture, the flow was 730 g/m^2^·h, and the selectivity was 13.

Thus, compared to the pervaporation separation index of isopropanol/water mixture with the known analog polyurethane membranes, the membranes obtained from AEPA-PEG have high values.

#### 3.1.4. Thermogravimetric Analysis of AEPA-PU

The detected difference in the regularities of changes in the conditions for the film material formation on the molecular structure of AEPA-PU also affects their thermal behavior. Thus, for AEPA-PU obtained at SC = 60 wt.%, the onset of weight loss in the air is observed at a temperature that, on average, is 7 °C higher than the temperature of the onset of weight loss for AEPA-PU obtained from the SC = 100 wt.% (Table 2). At the same time, the temperature corresponding to 50% weight loss (T_50%_) for AEPA-PU obtained from the SC = 100 wt.% exceeds by 10–30 °C that for AEPA-PU obtained at SC = 60 wt.%. For AEPA-PU obtained from the SC = 100 wt.%, a higher (by 2 wt.%) char residue content is also observed. An increase in the sample’s thermal stability in the high-temperature region can be a consequence of the ionomeric organophosphorus component clustering Table 2.

The fact that an increase in the SC content to 100 wt.% leads to the processes of clustering of the organophosphorus ionomer component in AEPA-PU is also confirmed by the data of TGA analysis carried out in an inert medium (Table 3). Thus, AEPA-PU obtained at SC = 100 wt.% turned out to be significantly more heat-resistant in comparison with AEPA-PU obtained at SC = 60 wt.%. The assumption about the clustering of ionomeric organophosphorus structures in AEPA-PU is also confirmed by the TGA data obtained for EPA-PU, which do not contain ionogenic groups (Table 3). In this case, an increase in SC from 60 to 100 wt.% during the production of EPA-PU does not lead to noticeable changes in the thermal stability of the samples.

#### 3.1.5. Thermomechanical Analysis of AEPA-PPG-1000-PU and EPA-PPG-1000-PU

**Figure 8:**
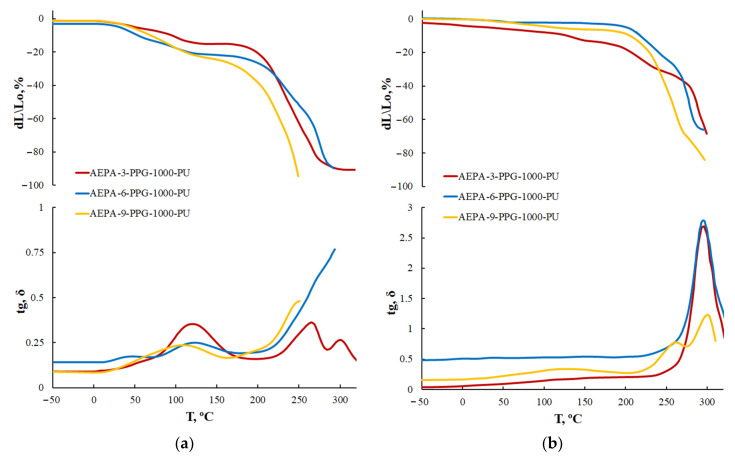
TMA curves and temperature dependence of the mechanical loss tangent (tg, σ) of AEPA-PPG-1000-PU, obtained at SC = 100 wt.% (**a**); SC = 60 wt.% (**b**).

For AEPA-PPG-1000-PU obtained at SC = 100 wt.%, two transitions are observed on the TMA and DMA curves. A low-intensity transition begins at 50 °C; a high-intensity transition begins at 100–130 °C (Figure 8). Transitions due to the destruction of supramolecular formations are not observed in this temperature range for AEPA-PPG-1000-PU obtained at SC = 60 wt.%. Judging by the higher thermal stability of the AEPA-PPG-1000-PU samples obtained at SC = 100 wt.% compared to the AEPA- PPG-1000-PU obtained at SC = 60 wt.%., it can be concluded that the supramolecular structures that break down at 100–130 °C are clusters resulting from the combination of phosphate anions in the AEPA-PPG-1000-PU matrix [42,43].

**Figure 9:**
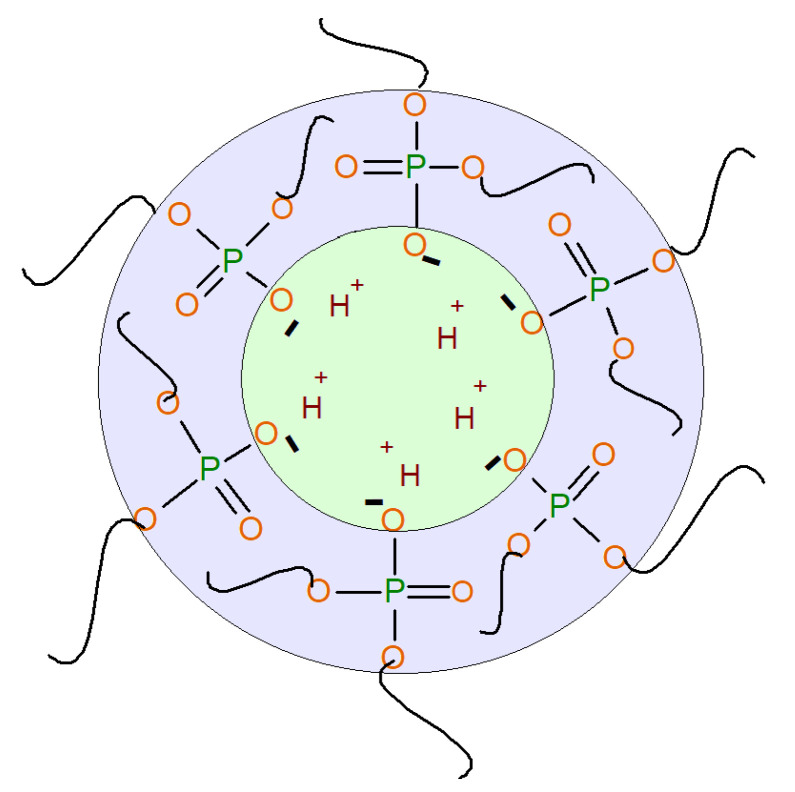
Scheme of the cluster structures formation in the AEPA-PPG-1000-PU.

Apparently, the increase in the concentration of the urethane-forming system based on AEPA-PPG-1000 and PIC up to the synthesis without the use of a solvent is due to intensive clustering processes with the participation of PO^−^ groups that present in AEPA-PPG-1000 (Figure 9).

**Figure 10:**
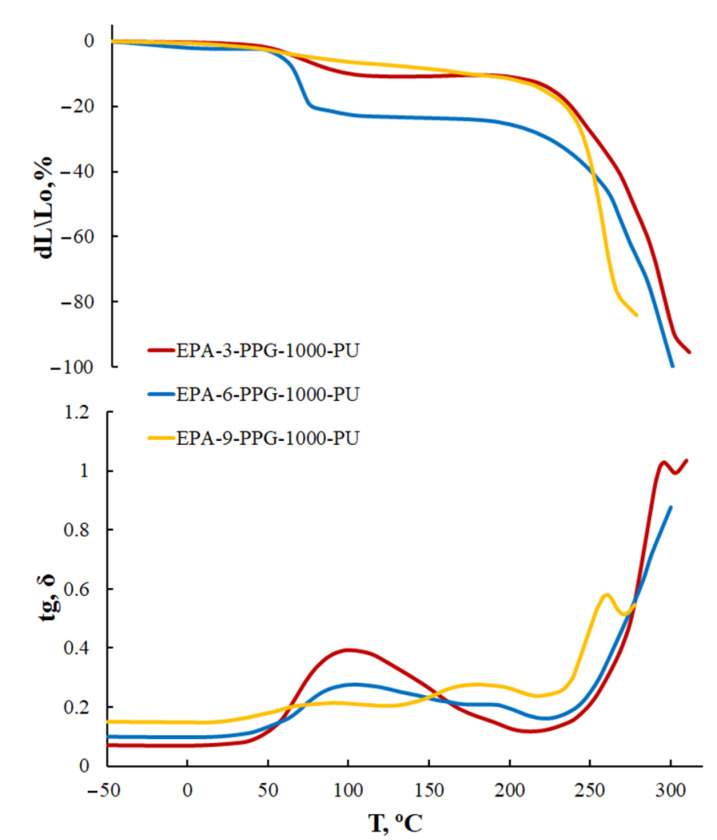
TMA curves and temperature dependence of the mechanical loss tangent (tg, σ) of EPA-PPG-1000-PU, obtained at SC = 100 wt.%.

In the case of EPA-PPG-1000-PU, polyphosphate structures are formed. On the TMA and DMA curves (Figure 10) in the region of 50 °C, a relaxation transition begins due to the clustering of polyphosphate structures. The lower temperature of decomposition of cluster structures for EPA-PPG-1000-PU compared to AEPA-PPG-1000-PU (located in the region of 100 °C) is due to the absence of phosphate anions in their composition, which combine during clustering through ionic interactions. For EPA-PPG-1000-PU, the intensity of relaxation transitions depends largely on the content of OPA in the EPA composition in comparison with AEPA. This circumstance confirms the conclusions drawn. Thus, in the case of EPA-3-PPG-1000-PU, the content of polyphosphates is still low, whereas, in EPA-9-PPG-1000-PU, it is so high that these structures become bulky and, apparently, no longer participate in clustering.

**Figure 11:**
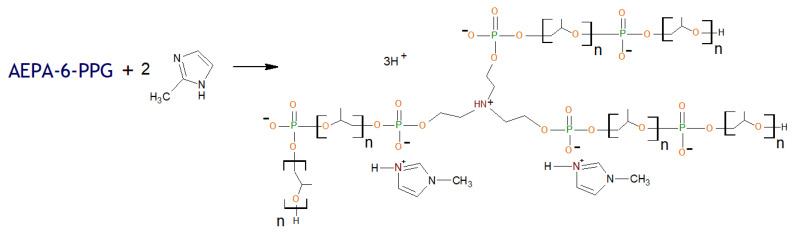
Formation of AEPA-6-PPG-MIA ([MIA]/[TEOA] = 2).

**Figure 12:**
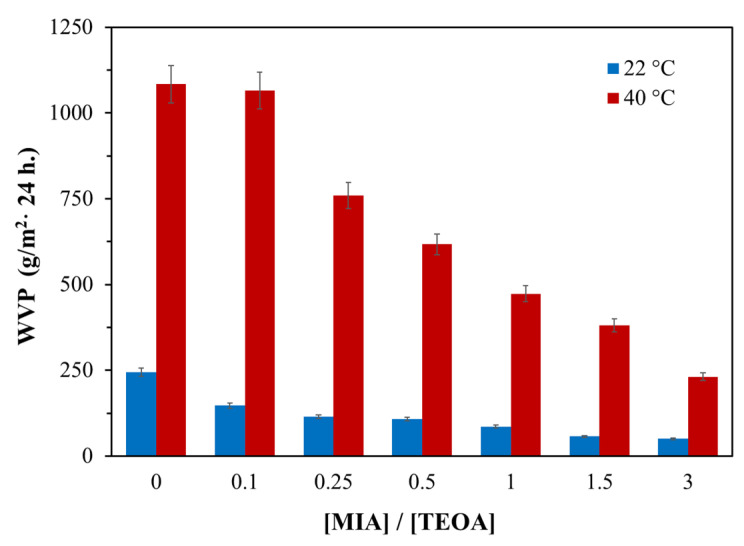
Water vapor permeability coefficients for AEPA-PPG-1000-MIA-PU obtained at SC = 100 wt.%, on molar ratio [MIA]/[TEOA].

To confirm that the clustering of phosphate anions is a decisive factor in the occurrence of vapor permeability of AEPA-PPG-1000-PU, AEPA-PPG-1000-MIA-PU were synthesized (Figure 11), and their vapor permeability was studied. Compared to the proton, 2-methylimidazole is incomparably large. Consequently, it seems difficult to create the necessary conditions for the clustering of phosphate anions in this case. Indeed, according to the data shown in Figure 12, for AEPA-PPG-1000-MIA-PU, a rapid decrease in vapor permeability values is observed with an increase in the content of 2-methylimidazole in such polyurethanes.

### 3.2. Membranes Based on AEPA-PEG-400-PU

**Figure 13:**
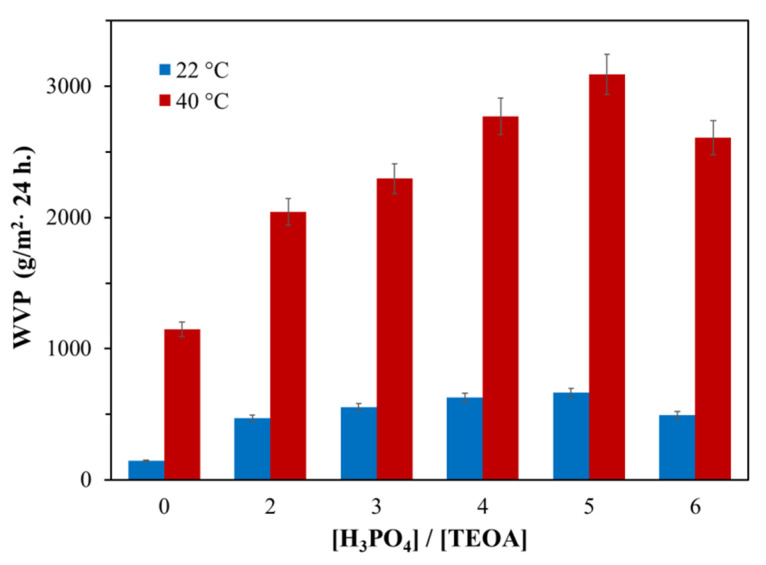
Water vapor permeability coefficients for AEPA-PEG-400-PU obtained at SC = 100 wt.%, on molar ratio [H_3_PO_4_]/[TEOA].

When polyether PPG-1000 was replaced by PEG-400 in the synthesis of AEPA-PEG-400-PU (SC = 100 wt.%), the vapor permeability of film samples increased more than three times (Figure 13). At the same time, the highest values of vapor permeability are observed for AEPA-5-PEG-400-PU.

An increase in water vapor permeability values is accompanied by a significant increase in the pervaporation characteristics of AEPA-PEG-400-PU compared to AEPA-PPG-1000-PU (Table 1). Similar to the increase in the water vapor permeability coefficient from AEPA-3-PEG-400-PU to AEPA-5-PEG-400-PU, a significant increase in the membrane performance is observed in the same series. With an increase in feed temperature, an increase in flux is accompanied by a regular decrease in selectivity values; however, high values of the pervaporation separation index are observed in all cases. The highest PSI values are achieved for AEPA-5-PEG-400-PU. Water adsorption of AEPA-5-PEG-400-PU (9.4 wt.%) significantly exceeds the corresponding values obtained for AEPA-5-PPG-1000-PU (3.4 wt.%).

Nevertheless, with an increase in the molar ratio of [H_3_PO_4_]/[TEOA] to 6, there is a slight drop in both the vapor permeability coefficient (Figure 13) and the pervaporation efficiency (Table 1) of obtained AEPA-6-PEG-400-PU. The observed effect can be attributed to the fact that an increase in the molar ratio [H_3_PO_4_]/[TEOA] to 6 leads to an increase in the probability of the formation of polyphosphate structures of a non-ionic nature in AEPA-6-PEG-400-PU. A decrease in the values of the vapor permeability coefficient and pervaporation efficiency is accompanied by a corresponding drop in the water absorption of the AEPA-6-PEG-400-PU samples to 7.6%.

#### 3.2.1. Surface-Active Properties of AEPA-PPG-400/1000 and AEPA-PEG-400

To explain such significant differences in the efficiency of water transport through membranes based on AEPA-PEG-400-PU and AEPA-PPG-400/1000-PU, comparative studies were made from the point of view of the influence of the molecular weight, hydrophilicity, and hydrophobicity of the polyethers used on the values of the viscosity and density and surface-active properties of AEPA-PEG-400-PU and AEPA-PPG-400/1000-PU, which are ionic polyol components in the synthesis of the studied polyurethanes.

Thus, for PEG-400, the critical micelle concentration (Figure 7c) exceeds the CMC for PPG-1000 (Figure 7b) by 100 times, and the lowest values of surface tension for PEG-400 reach 55 mN/m and are lower in comparison with the lowest σ values observed for PPG-1000 (62 mN/m). Such a large difference in the surfactant properties of PEG-400 and PPG-1000 has a significant effect on the supramolecular organization of AEPA-PEG-400 and AEPA-PPG-1000. Thus, by observing the regularities of changes in surface-active properties, we can determine that AEPA-PPG-1000 exhibits a greater tendency to micelle formation than AEPA-PEG-400.

In order to exclude the influence of molecular weight on the difference in the manifestation of the surface-active properties of AEPA-PEG-400 and AEPA-PPG-1000, similar measurements were carried out for AEPA-PPG-400 (Figure 7d). In this case, the surface-active properties of PPG-400 are similar to those of PPG-1000. However, due to the shorter length of the polypropylene component, the surface tension values of AEPA-PPG-400 are higher than AEPA-PPG-1000 (Figure 7b).

#### 3.2.2. Study of the Viscosity and Density of AEPA-PPG-400/1000 and AEPA-PEG-400

**Figure 14:**
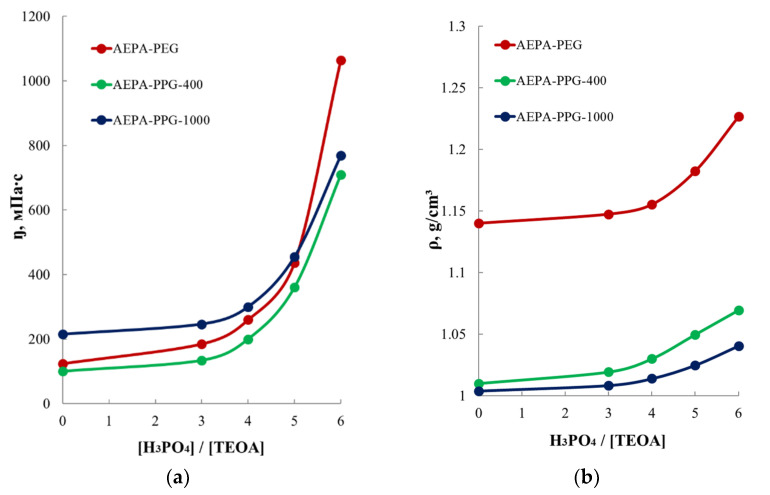
Dependence of dynamic viscosity (**a**) and density (**b**) of AEPA-PEG-400 and AEPA-PPG-400/1000 on molar ratio [H_3_PO_4_]:[TEOA]. T = 20 °C.

The fact that the nature of polyether affects the supramolecular organization of the corresponding AEPA can also be observed from the regularities of changes in the values of the viscosity and density of aminoethers of *ortho*-phosphoric acid. According to Figure 14, the density of AEPA-6-PEG-400 at T = 20 °C relative to the density of PEG-400 increases by almost 0.2 g/cm^3^, whereas the density of AEPA-6-PPG-400 relative to the density of PPG-400 and AEPA-PPG-1000 relative to the density of PPG-1000 increases only by 0.05 g/cm^3^ and 0.03 g/cm^3^, respectively. The density with an increase in the content of ionomeric structures also increases for AEPA-PEG-400-PU (Table 4). Similar patterns are observed for the values of the dynamic viscosity of the studied aminoethers of *ortho*-phosphoric acid (Figure 14). Thus, the dynamic viscosity of AEPA-6-PEG-400 at T = 20 °C relative to the dynamic viscosity of PEG-400 increases almost six times, while the dynamic viscosity of AEPA-6-PPG-1000 relative to the dynamic viscosity of PPG-1000 increases only three times.

It should be noted that the density of PPG-1000 is noticeably lower than that of PEG-400. At the same time, the surface tension is lower, and the critical micelle concentration is higher for PEG-400 compared to PPG-400. These circumstances lead to different manifestations of surface-active properties, density, and viscosity of AEPA obtained on their basis. As a result, AEPA-PPG-1000 is characterized by the active combination of phosphate anions into clusters that are collected in ion channels surrounded by macro chains of polypropylene glycol-1000. The hydrophobicity of PPG-1000 makes it difficult for water to reach ion channels and move it through the polymer matrix. In the case of AEPA-PEG-400, the tendency to form micellar structures is markedly reduced, which leads to a decrease in the probability of the formation of ionic clusters. At the same time, due to the continuity of the hydrophilic polymer matrix, polyurethanes based on PEG-400 and PIC (PEG-400-PU) are water vapor permeable. The presence of ionic groups in the composition of AEPA-PEG-400-PU increases the mobility of water molecules in such polyurethanes.

#### 3.2.3. Thermogravimetric Analysis of AEPA-PEG-400-PU

The conclusions drawn are confirmed by a comparative analysis of the thermal stability of AEPA-PPG-1000-PU and AEPA-PEG-400-PU. As shown above, using the example of AEPA-PPG-1000-PU (Table 2 and Table 3), the formation of ionic clusters is accompanied by an increase in the thermal resistance of polyurethanes with an increase in their content of the ionic component. In the case of AEPA-PEG-400-PU, an increase in the content of phosphate anions in polyurethanes is not accompanied by an increase in their thermal stability (Table 5).

#### 3.2.4. Thermomechanical Analysis of AEPA-PEG-400-PU

**Figure 15:**
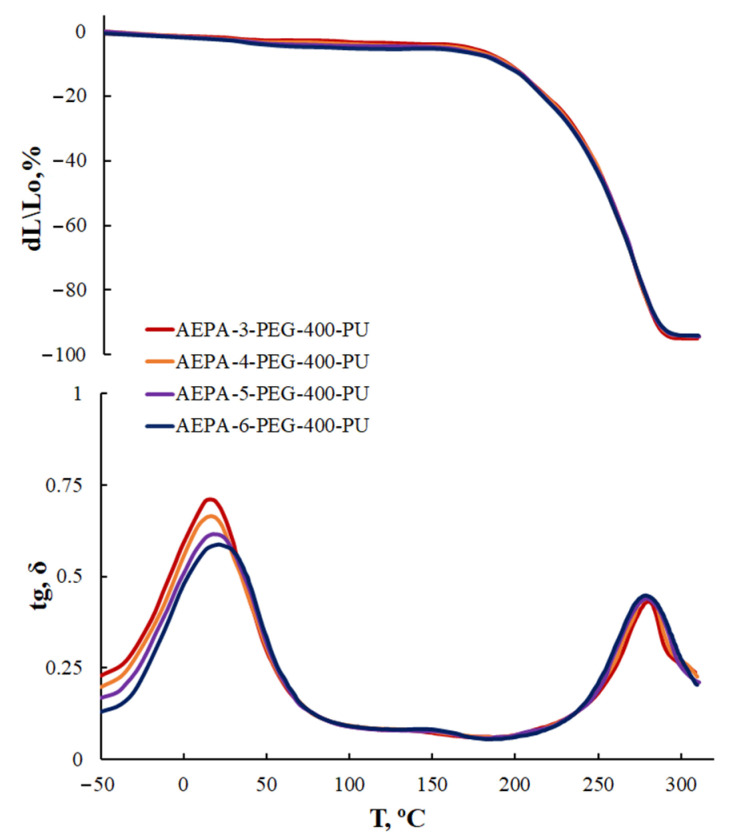
TMA curves and temperature dependence of the mechanical loss tangent (tg, σ) of AEPA-PEG-400-PU, obtained at SC = 100 wt.%.

In addition, the curves of thermomechanical and dynamic mechanical analysis do not show transitions in the range of 100–130 °C, typical for AEPA-PPG-1000-PU (Figure 15).

#### 3.2.5. Tensile Stress–Strain Analysis of AEPA-PEG-400-PU

**Figure 16:**
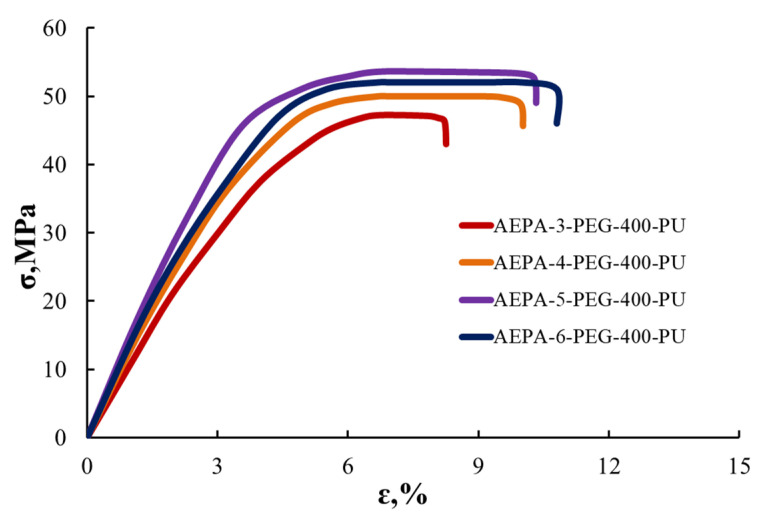
Tensile tests of AEPA-PEG-400-PU, obtained at SC = 100 wt.%.

According to Figure 16, samples of polyurethane membranes based on AEPA-PEG-400-PU, exhibiting the highest values of vapor permeability and pervaporation characteristics when separating isopropanol/water mixtures, are the most durable. The high strength and heat resistance of AEPA-PEG-400-PU make such polyurethanes promising membrane materials for the pervaporation separation of isopropanol/water mixtures.

## 4. Conclusions

In this present work, based on aminoethers of *ortho*-phosphoric acid synthesized using polyethers of hydrophilic (PEG) and hydrophobic (PPG) nature as lateral flexible chain branches, polyurethane ionomers were obtained as water vapor permeable and pervaporation membranes. From the results obtained in this work, it can be inferred that the water vapor permeability and pervaporation separation index for isopropanol/water mixtures significantly increase by the rising of the content of H_3_PO_4_ in the AEPA-PPG-1000-PU composition and is connected with the clustering of phosphate anions. However, the hydrophobicity of the polypropylene glycol surrounding the clusters makes it difficult for water to move through the polymer matrix. Due to the hydrophilicity of polyethylene glycol, high values of water vapor permeability and pervaporation separation index are achieved for AEPA-PEG-400-PU. As in the case of AEPA-PPG-1000-PU, in AEPA-PEG-400-PU, the water vapor permeability and membrane performance noticeably increase with the rising of the content of ionogenic phosphate anions in the obtained polyurethanes.

To substantiate the key role of ionogenic phosphate anions in the formation of water vapor permeability of AEPA-PPG-1000-PU, polyurethanes of a non-ionic nature (EPA-PPG-1000-PU) were studied. The water vapor permeability of EPA-PPG-1000-PU also increases with an increase of H_3_PO_4_ content in the composition of EPA. However, the water vapor permeability coefficient values themselves turned out to be more than two times lower compared to the samples of AEPA-PPG-1000-PU.

To confirm that clustering of phosphate anions is a decisive factor in the occurrence of vapor permeability of AEPA-PPG-1000-PU, AEPA-PPG-1000-MIA-PU were synthesized, and their vapor permeability was studied. Compared to the proton, 2-methylimidazole is incomparably large. Consequently, in this case, it seems difficult to create conditions for the clustering of phosphate anions. A rapid decrease in vapor permeability values is observed with an increase in the content of 2-methylimidazole in such polyurethanes. The assumption about the clustering of ionomeric organophosphorus structures in AEPA-PU is also confirmed by the TGA data, thermomechanical analysis, and mechanical loss tangent measurements.

It was found that clustering in polyurethane ionomers is due to the peculiarities of the chemical and supramolecular structure of AEPA. In order to confirm the decisive role of the supramolecular structure on the vapor permeability of AEPA-PPG-1000-PU, polyurethanes based on AEPA-PPG-2000 were synthesized. An increase in the molecular weight of PPG led to a decrease in the water vapor permeability coefficient for AEPA-PPG-2000-PU relative to AEPA-PPG-1000-PU.

By comparing the pervaporation separation index of isopropanol/water mixtures with the known analog polyurethane membranes, we find that the membranes obtained from AEPA-PEG have high values. The high strength and heat resistance of AEPA-PEG-400-PU make such polyurethanes promising membrane materials for the pervaporation separation of isopropanol/water mixtures.

## Figures and Tables

**Table 1:** Isopropanol dehydration using pervaporation membranes based on AEPA-PPG-1000-PU and AEPA-PEG-PU, obtained at SC = 100 wt.%. Isopropanol in the feed, 85 wt.%.

Polyurethane	Water in Permeate, wt.%	Flux, g/m^2^·h	Separation Factor	PSI, g/m^2^·h
**Feed temperature, 40 °C**				
AEPA-3-PPG-1000-PU	84.4	880	51	44
AEPA-4-PPG-1000-PU	83.2	1021	49	49
AEPA-5-PPG-1000-PU	82.8	1145	52	58.4
AEPA-6-PPG-1000-PU	80.9	1250	45	52.8
AEPA-3-PEG-400-PU	96.4	653	151	98.0
AEPA-4-PEG-400-PU	96.3	805	148	118.3
AEPA-5-PEG-400-PU	90.9	2039	56	112.2
AEPA-6-PEG-400-PU	90.1	1580	65	101.1
**Feed temperature, 60 °C**				
AEPA-3-PPG-1000-PU	83.0	1800	28	48.6
AEPA-4-PPG-1000-PU	82.0	2551	26	56.3
AEPA-5-PPG-1000-PU	81.1	2655	24	74.3
AEPA-6-PPG-1000-PU	79.1	2853	21	57.1
AEPA-3-PEG-400-PU	92.7	1859	72	132.0
AEPA-4-PEG-400-PU	92.6	2298	71	161.0
AEPA-5-PEG-400-PU	90.5	3734	54	197.9
AEPA-6-PEG-400-PU	89.2	2624	62	160.0

**Table 2:** Characteristics of the thermal stability of AEPA-PPG-1000-PU under air atmosphere.

Polyurethane *	T_5%_ (°C)	T_10%_ (°C)	T_50%_ (°C)	Coke Content at 600 °C, wt.%
AEPA-3-PPG-1000-PU	278/271	292/284	369/394	5/2
AEPA-4-PPG-1000-PU	275/266	292/283	381/414	3.5/6
AEPA-5-PPG-1000-PU	277/268	293/282	378/410	4/6
AEPA-6-PPG-1000-PU	280/263	294/284	383/401	5/6

* The values in the numerator refer to SC = 60 wt.%, and the values in the denominator refer to SC = 100 wt.%.

**Table 3:** Characteristics of the thermal stability of AEPA-PPG-1000-PU and EPA-PGG-1000-PU under nitrogen atmosphere.

Polyurethane *	T_5_% (°C)	T_10_% (°C)	T_50_% (°C)	Coke Content at 600 °C, wt.%
AEPA-3-PPG-1000-PU	308/296	322/313	362/364	17/20
AEPA-4-PPG-1000-PU	235/295	268/309	310/352	16/19.1
AEPA-5-PPG-1000-PU	220/291	243/308	300/349	14/18.5
AEPA-6-PPG-1000-PU	275/294	300/312	350/350	15/16
EPA-3-PPG-1000-PU	300/298	317/318	365/372	18/26
EPA-4-PPG-1000-PU	295/298	311/316	355/368	17/21
EPA-5-PPG-1000-PU	280/296	301/318	350/359	16/16.7
EPA-6-PPG-1000-PU	280/282	300/300	350/350	17/16.3

* The values in the numerator refer to SC = 60 wt.%, and the values in the denominator refer to SC = 100 wt.%.

**Table 4:** Density of AEPA-PEG-400-PU.

Polyurethane	Density, g/cm^3^
AEPA-3-PEG-400-PU	1.193
AEPA-4-PEG-400-PU	1.194
AEPA-5-PEG-400-PU	1.223
AEPA-6-PEG-400-PU	1.229
AEPA-3-PPG-1000-PU	1.175
AEPA-4-PPG-1000-PU	1.184
AEPA-5-PPG-1000-PU	1.185
AEPA-6-PPG-1000-PU	1.189

**Table 5:** Characteristics of the thermal stability of AEPA-PEG-400-PU (SC = 100 wt.%) under a nitrogen atmosphere.

Polyurethane	T_5_% (°C)	T_10_% (°C)	T_50_% (°C)	Coke Content at 600 °C, wt.%
AEPA-3-PEG-400-PU	291	315	383	26.5
AEPA-4-PEG-400-PU	292	316	385	27.5
AEPA-5-PEG-400-PU	296	313	390	28.5
AEPA-6-PEG-400-PU	290	309	398	30.0

## Data Availability

The data presented in this study are available on request from the corresponding author.
